# An Evaluation of Heart Rate Variability in Female Youth Soccer Players Following Soccer Heading: A Pilot Study

**DOI:** 10.3390/sports7110229

**Published:** 2019-11-04

**Authors:** Alexandra B. Harriss, Kolten Abbott, Kurt Kimpinski, Jeffrey D. Holmes, Andrew M. Johnson, David M. Walton, James P. Dickey

**Affiliations:** 1 School of Kinesiology, University of Western Ontario, London, ON N6A 3K7, Canada; aharri73@uwo.ca (A.B.H.); kolten.abbott@gmail.com (K.A.); 2 School of Physical Therapy, University of Western Ontario, London, ON N6A 3K7, Canada; 3 Department of Clinical Neurological Sciences, The University of Western Ontario, London, ON N6A 3K7, Canada; kkimpin@uwo.ca; 4 School of Occupational Therapy, University of Western Ontario, London, ON N6A 3K7, Canada; jholme@uwo.ca; 5 School of Health Studies, University of Western Ontario, London, ON N6A 3K7, Canada; ajohnson@uwo.ca

**Keywords:** concussion, subconcussion, autonomic nervous system, adolescents, head impacts

## Abstract

Most head impacts in soccer occur from purposeful heading; however, the link between heading and neurological impairment is unknown. Previous work suggests concussion may result in an uncoupling between the autonomic nervous system and cardiovascular system. Accordingly, heart rate variability (HRV) may be a sensitive measure to provide meaningful information regarding repetitive heading in soccer. The purpose of this pilot study assesses the feasibility of measuring HRV to evaluate autonomic function following soccer heading. Sixteen youth female participants underwent heart rate monitoring during a heading and footing condition. Participants completed a five minute resting supine trial at the start and end of each testing session. Standard 450 g soccer balls were projected at 6 m/s towards participants. Participants performed five headers, for the header condition, and five footers for the footer condition. The HRV for resting supine trials, pre- and post-header and footer conditions were assessed for both time and frequency domains. HRV effect sizes were small when comparing conditions, except absolute low frequency (*d* = 0.61) and standard deviation of the normal-normal (NN) intervals (*d* = 0.63). Participant retention and adherence were high, without adverse events. Findings suggest HRV is a feasible measure for evaluating the effects of heading on autonomic function.

## 1. Introduction

While youth sport-related concussions are both a healthcare [1] and societal [2] concern, the majority of head impacts in sport are subconcussive [3,4]. A subconcussive impact is an impact to the head that does not result in clinical signs or symptoms of concussion [5]; however, long-term exposure may lead to neurological impairment [6]. One study has reported that soccer head impacts can be as large as 113 g, which has a greater than 90% risk of concussion according to a published risk curve [7], yet that study found no evidence of concussion [4]. In soccer, the majority of head impacts result from purposeful headers [8]. Given that youth’s brains are still developing [9] and are vulnerable to risk of injury from soccer heading, it is therefore important to quantify heading risk among youth players. Nevertheless, little research has investigated the effects of soccer heading in this age group [10,11,12].

The accumulation of header impacts and their influence on brain health have been investigated by various research groups; however, findings remain inconclusive [13,14,15]. This may suggest that existing measures of neurophysiological function are insensitive to detect changes following soccer heading, and that a more sensitive measure may be required. The autonomic nervous system (ANS) influences the cardiovascular system [16], and is impaired in patients with brain injury [17]. Therefore, it may be a fruitful domain in which to measure subconcussive impacts.

The cardiovascular system is influenced by various physiologic factors; however, the autonomic nervous system (ANS) is the most prominent [16]. The ANS is comprised of two divisions: parasympathetic and sympathetic. Fluctuations in heart rate indicate the interplay between these two systems such that increases in parasympathetic activity decrease heart rate, and increases in sympathetic activity increase heart rate [16]. Heart rate variability (HRV) is the variation in time intervals between heartbeats and has been used to quantify autonomic function of the parasympathetic and sympathetic nervous system. For example, patients with brain injury show reductions in power spectral content of the HRV signal [17]. Patients with mild traumatic brain injury (mTBI) [18], and sport-related concussion [19,20], show increased sympathetic activity and decreased parasympathetic activity. This autonomic dysregulation following head injury suggests that the ANS is not modulating heart rate appropriately, and may reflect autonomic and cardiovascular uncoupling [20]. Accordingly, HRV may provide meaningful information about the heart–brain relationship, particularly sympathetic and parasympathetic nervous system activity, following subconcussion and concussion injury.

Youth soccer players are vulnerable to risk of injury from soccer heading as their brains are still developing [9]. Female youths seem to be particularly vulnerable based on their increased rates of concussion [21]. To date, no studies have evaluated the feasibility of assessing HRV in youth soccer. Accordingly, the objective of this investigation was to conduct a pilot study in female youth soccer players, in accordance with current guidelines [22], to investigate (1) the feasibility of the study design (participant recruitment, adherence, retention, and adverse events) and (2) the effect sizes associated with changes in HRV indices between a soccer heading condition and control footer condition. These data will help provide valuable information for future investigations regarding acceptable heading thresholds in youth soccer.

## 2. Materials and Methods

### 2.1. Participants

A convenience sample of 16 female soccer players (12.5 ± 0.5 years; 49.1 ± 5.5 kg; 1.6 ± 0.1 m) on one female soccer team competing in the Ontario Player Development League was used for this study. All participants were actively engaged in their soccer season at the time of testing. Participants played in approximately one game and four practices per week over their 20 week soccer season. Participants were excluded if they had any neurological, cardiac, or psychiatric conditions. Two participants suffered a concussion more than two years earlier, and were both medically cleared by a physician. Written informed consent from participants and parents was obtained prior to participation. The study was approved by the Health Sciences Research Ethics Review Board (REB protocol 107948) at the University of Western Ontario.

### 2.2. Protocol

Participants completed two testing conditions separated by six weeks. All participants performed the header condition first and a footer (control) condition second. Testing took place at the start of the teams’ outdoor practice at 18:40. Participants did not perform strenuous physical exercise 24 h prior to testing due to its influence on autonomic modulation.

Firstbeat heart rate monitors (Firstbeat Technologies Ltd., Jyväskylä, Finland) were worn across the chest at the level of the xiphoid to record measures of the normal-normal (NN) intervals with 1 ms resolution. Participants also wore a headband instrumented with a head impact sensor (GFT2, Artaflex Inc., Markham, ON, Canada) positioned at the occipital bone [8]. The GFT2 contains a triaxial accelerometer to measure linear head acceleration (1 g resolution), and rotational velocity (1°/s resolution). For this study a 7 g threshold was used to initiate header impact data collection.

All participants completed a quiet five-minute resting supine trial at the start of each testing session to measure baseline HRV. Participants were encouraged to breathe normally and refrain from talking or moving during resting trials. After each participant completed their header or footer condition, they performed another five-minute resting supine trial.

All conditions involved projecting standard 450 g soccer balls (inflated to 8 psi) at 6 m/s towards participants, from a distance of 8 m, using a mechanical soccer ball pitching machine (Pro Trainer Soccer, Alameda, CA, USA). For the header condition, each participant performed five headers, separated by 10 s intervals. For the footer (control) condition, each participant used their foot to volley the ball five times, separated by 10 s intervals (to match the header condition).

### 2.3. Data Analysis

A primary outcome measure of interest was feasibility, encompassing participant recruitment, retention, and adherence. Recruitment was assessed by the number of successfully recruited participants from the 18 player roster. Retention was assessed by the proportion of participants who attended both testing conditions. Reasons for not attending the sessions were recorded. Adherence was assessed by the number of participants who wore the wireless headband sensors and the heart rate monitor. Any adverse events reported during testing were recorded.

Analyses of linear HRV metrics in the time and frequency domains were completed using Kubios HRV software (Version 2.0, Biosignal Analysis and Medical Imaging Group, Kupio, Finland). The HRV data were manually inspected and edited for ectopic beats [16] and were assessed during the resting supine trials, pre- and post-header and footer conditions. Time domain parameters included the standard deviation of the NN intervals (SDNN), the root mean square of successive NN interval differences (RMSSD), and the proportion of consecutive NN intervals that differed by more than 50 ms (pNN50). A power spectral density analysis was used with a non-parametric method (Fast Fourier Transform based Welch’s periodogram) with a high frequency band set at 0.15–0.4 Hz, and low frequency band set at 0.04–0.15 Hz [23]. The high frequency and low frequency power were reported in units of absolute values of power (ms^2^) and normalized units (nu). The ratio between low frequency and high frequency power (LF/HF) was also calculated. The peak linear acceleration and peak angular velocity data were recorded for header impacts.

Descriptive statistics were used to present participant characteristics and feasibility data. Head impact data are reported as mean ± standard deviation. HRV data for all conditions are reported as the mean ± standard deviation and 95% confidence interval (CI). The difference for headers (Δheader = post–pre), as well as the difference for footers (Δfooter = post–pre) were calculated for all time and frequency domain parameters. The within-group change scores for each condition (mean ± standard deviation), 95% CI, and effect sizes are reported. Effect sizes were calculated using Cohen’s *d*, interpreted as trivial (0–0.19), small (0.2–0.49), medium (0.5–0.79), and large (0.8 and greater). Probability-based inferential statistics are not reported in accordance with pilot study guidelines.

## 3. Results

Of the sixteen female players recruited for the soccer team (89% recruitment), all participants consented to wear the heart rate monitor, whereas only seven of these participants (44%) consented to wear the headband sensor. In terms of participant retention, 15 participants completed both testing sessions (94% retention). One participant did not attend the second session because of a conflict with an academic commitment, and subsequently was removed from HRV analysis. No adverse events were reported; however, one participant had poor electrode contact during header HRV recordings and was removed from HRV analysis.

The average peak linear head acceleration and angular velocity for headers was 16.6 g ± 3.6 g and 531.5 ± 161.7°/s respectively. The pre- and post-header and footer HRV indices are reported for time and frequency domain outcome measures in Table 1. The mean change and effect sizes for time and frequency domain measures are reported in Table 2. For all time domain measures, effect sizes between ∆header and ∆footer were small, except SDNN (*d =* 0.63). SDNN increased following both the header (11.2 ± 22.1 ms, 95% CI −1.6, 24) and footer condition (31.6 ± 31.8 ms, 95% CI 13.2, 49.9). Effect sizes for all frequency domain measures were small, except absolute low frequency power (*d* = 0.61) (Table 2). Absolute low frequency power increased for both the header (219.6 ± 875.3 ms^2^, 95% CI −285.7, 725.0), and footer conditions (829.5 ± 1186.0 ms^2^, 95% CI 144.7, 1514.3).

## 4. Discussion

This pilot study investigated the feasibility of using heart rate monitors and headband sensors in female youth soccer. As well the effectiveness of using HRV parameters to evaluate changes in autonomic function following heading compared to control footer conditions. Our results suggest that HRV is a feasible outcome measure that may provide meaningful information to assess heading thresholds in youth soccer.

Recruitment was shown to be highly feasible; we successfully recruited all players that attended soccer practice. While all participants agreed to wear the heart rate monitor, only 44% of these participants consented to wear the headband sensor. This was likely because the head sensors were part of a larger scale study and these participants were already accustomed to wearing the head sensor technology. Because HRV indices were the main outcome measure, this low adherence for head impact sensors was not concerning, but should be considered for studies that measure head impact accelerations in youth soccer. Similarly, retention was excellent, as only one player could not attend the second session. No adverse advents were reported.

Time and frequency domain indices of the HRV signal did not suggest autonomic dysregulation during heading as compared to the footer condition. Although effect size findings indicate meaningful differences in some HRV outcomes, such as SDNN and absolute low frequency power, these changes are not indicative of autonomic impairment. Reduced HRV has been observed in patients with concussion [7,8,9], which may be related to an uncoupling between the cardiovascular system and ANS. In this study, we did not observe this uncoupling in response to five consecutive headers. Our observations were, however, limited to resting supine conditions. Some studies have shown changes with exercise exertion but not at rest [20]. Therefore, changes associated with repetitive heading may be evident under an autonomic challenge condition. 

For head conditions, the same ball trajectory and speed were used for all participants. The relatively low variability between header impact accelerations suggests that the head impacts measured from the seven instrumented players reflect the heading exposures for the entire group. While the present study shows no evidence of autonomic dysregulation following five headers, autonomic impairments are related to the severity of head injury [17]. Accordingly, the head impact accelerations experienced from five headers in this study may be too mild to result in any detectable changes in autonomic function at resting conditions. The number of headers performed in this study reflects the typical heading exposure [24] and head accelerations in female youth soccer [25]. However, we do not know whether a greater number of headers or larger head impact accelerations would show transient changes in autonomic function.

We did not control for respiratory rate, which can influence HRV. However, participants were encouraged to breathe normally, and given the within-subject study design we believe the current findings are not heavily influenced by this measure as any bias would have been systematic between conditions. In addition, due to participant availability, testing days were separated by six weeks. All players were on the same soccer team and their baseline heart rates were similar for both testing conditions. One limitation of this study is that we only evaluate youth female players; however, investigating female youth soccer players is significantly relevant as females may be more susceptible to concussion than males [21]. In addition, due to the pilot study design, and small sample size we are unable to assess whether the head impacts that players incurred over the study period negatively influenced either parasympathetic or sympathetic nervous system functioning.

Eliminating or restricting heading in female youth soccer may help to reduce the cumulative head impact burden, and possible neurological sequelae resulting from heading. Youth’s brains are still developing [9], and may be more vulnerable to cumulative soccer heading. Data should drive decisions about heading regulations, and therefore it is important that future studies are sufficiently powered to enable definitive conclusions. Our pilot study data suggests that the sample sizes depends on the outcome measure. For example, accounting for a 6% dropout, a sample size of 23 participants would be required for SDNN, and 25 participants for absolute low frequency power, to achieve 80% power for detecting a difference between conditions with 95% confidence. If other metrics were desirable, considerably larger samples would be required.

In conclusion, our study shows that the analysis of HRV is a feasible measure in female youth soccer as well as participant retention to evaluate the influence of soccer heading on sympathetic and parasympathetic nervous system activity. The findings of this pilot study provide critical information for those wishing to conduct mechanistic studies into the effects of soccer heading impacts on neural or autonomic function in female youth soccer players. Heart rate variability may be adequately sensitive to identify effects in short term testing conditions. Changes in HRV analyses are demonstrated following concussion injury in youth [26]. Accordingly, future work should explore the importance of HRV measures and the potential influence of cumulative header impacts on brain health.

## Figures and Tables

**Table 1:** Changes in time domain and frequency measures following header and footer conditions.

Parameter	Post-Footer Poster Footer		Poster Footer		Pre-Header		Post-Header	
	Mean ± SD	CI	Mean ± SD	CI	Mean ± SD	CI	Mean ± SD	CI
**Heart Rate (bpm)**	77.2 ± 12.1	70.2–84.2	76.5 ± 12.0	69.5–83.5	76.8 ± 5.6	73.6–80.1	80.3 ± 8.3	75.5–85.1
**SDNN (ms)**	59.5 ± 30.0	42.1–76.7	91.0 ± 30.3	73.5–108.5	62.3 ± 22.9	49.0–75.5	73.5 ± 31.6	55.2–61.7
**RMSSD (ms)**	61.1 ± 35.9	40.3–81.8	67.3 ± 29.2	50.5-84.2	64.8 ± 34.8	44.7–84.8	62.0 ± 33.7	42.6–81.4
**pNN50 (%)**	31.4 ± 22.3	18.5–44.3	35.0 ± 16.6	25.4–44.6	32.2 ± 23.8	18.4–45.9	28.6 ± 18.7	17.9–39.4
**Total Power (ms^2^)**	3486.3 ± 3300.6	1580.6–5392.0	5659.1 ± 4348.6	3148.3–8170.0	3594.2 ± 2586.3	2100.9–5087.5	5358.1 ± 4883.6	2538.3–8177.8
**HF (ms^2^)**	1708.9 ± 1860.4	634.7–2783.0	1857.5 ± 2109.3	639.6–3075.4	1590.4 ± 1561.7	688.7–2492.1	1481.0 ± 1773.6	457.0–2505.0
**LF (ms^2^)**	893.6 ± 741.4	465.6–1321.7	1723.1 ± 1429.5	897.7–2548.5	969.5 ± 630.9	605.2–1333.8	1189.1 ± 903.8	667.3–1711.0
**HF (nu)**	58.6 ± 18.8	47.7–69.4	50.0 ± 15.6	40.9–59.0	52.3 ± 16.8	42.6–62.0	47.3 ± 17.5	37.2–57.4
**LF (nu)**	41.0 ± 18.5	30.3–51.7	49.7 ± 15.6	40.7–58.7	44.1 ± 15.9	34.9–53.3	52.3 ± 17.7	42.1–62.6
**LF/HF**	0.9 ± 0.9	0.4–1.5	1.2 ± 0.7	0.8–1.6	1.3 ± 1.3	0.5–2.0	1.4 ± 1.0	0.9–2.0

Note. SD: standard deviation; CI: confidence interval; SDNN: standard deviation of the NN intervals; RMSSD: root mean square of successive NN interval differences; PNN50: proportion of consecutive NN intervals that differed by more than 50 ms; HF (ms^2^): absolute high frequency; LF (ms^2^): absolute low frequency; LF (nu): normalized low frequency; HF (nu): normalized high frequency; LF/HF: ratio between low frequency and high frequency power.

**Table 2:** Mean change in time domain and frequency domain measures for pre- and post- header and footer conditions.

Parameter	∆ Footer		∆Header		Effect Size (*d*)
	Mean ± SD	CI	Mean ± SD	CI	
**ΔHeart Rate**	−0.7 ± 8.5	−5.6–4.3	3.4 ± 4.5	0.9–6.0	0.43
**ΔSDNN (ms)**	31.6 ± 31.8	13.2–49.9	11.2 ± 22.1	−1.6–24.0	0.63
**ΔRMSSD (ms)**	6.2 ± 26.8	−9.3–21.7	−2.8 ± 19.8	−14.2–8.6	0.25
**Δ pNN50 (%)**	3.6 ± 20.7	−8.4–15.5	−3.5 ± 11.2	−10.0–3.0	0.31
**ΔTotal Power (ms^2^)**	2172.9 ± 4951.2	−685.9–5031.6	1763.9 ± 4310.3	−724.9–4252.6	0.15
**ΔHF(ms^2^)**	148.6 ± 2511.6	−1301.5–1598.8	−109.4 ± 1331.2	−878.0–659.2	0.14
**ΔLF(ms^2^)**	829.5 ± 1186.0	144.7–1514.3	219.6 ± 875.3	−285.7–725.0	0.61
**ΔHF (nu)**	−8.6 ± 19.89	−20.1–2.8	−5.01 ± 13.41	−12.8–2.7	0.15
**ΔLF (nu)**	8.7 ± 19.5	−2.6–20.0	8.2 ± 16.4	−1.2–17.7	0.02
**ΔLF/HF**	0.2 ± 0.9	−0.3–0.8	0.2 ± 0.9	−0.3–0.7	0.06

Note. SD: standard deviation; CI: confidence interval; SDNN: standard deviation of the NN intervals; RMSSD: root mean square of successive NN interval differences; PNN50: proportion of consecutive NN intervals that differed by more than 50 ms; HF (ms^2^): absolute high frequency; LF (ms^2^): absolute low frequency; LF (nu): normalized low frequency; HF (nu): normalized high frequency; LF/HF: ratio between low frequency and high frequency power.
